# The data of the Swedish Malaise Trap Project, a countrywide inventory of Sweden's insect fauna

**DOI:** 10.3897/BDJ.8.e56286

**Published:** 2020-10-23

**Authors:** Dave Karlsson, Mattias Forshage, Kevin Holston, Fredrik Ronquist

**Affiliations:** 1 Station Linné, Färjestaden, Sweden Station Linné Färjestaden Sweden; 2 Swedish Museum of Natural History, Stockholm, Sweden Swedish Museum of Natural History Stockholm Sweden

**Keywords:** Malaise trap, insect fauna, inventory, survey, all-taxa biodiversity inventory, ATBI, Diptera, Hymenoptera

## Abstract

**Background:**

Despite Sweden's strong entomological tradition, large portions of its insect fauna remain poorly known. As part of the Swedish Taxonomy Initiative, launched in 2002 to document all multi-cellular species occurring in the country, the first taxonomically-broad inventory of the country's insect fauna was initiated, the Swedish Malaise Trap Project (SMTP). In total, 73 Malaise traps were deployed at 55 localities representing a wide range of habitats across the country. Most traps were run continuously from 2003 to 2006 or for a substantial part of that time period. The total catch is estimated to contain 20 million insects, distributed over 1,919 samples (Karlsson et al. 2020). The samples have been sorted into more than 300 taxonomic units, which are made available for expert identification. Thus far, more than 100 taxonomists have been involved in identifying the sorted material, recording the presence of 4,000 species. One third of these had not been recorded from Sweden before and 700 have tentatively been identified as new to science.

**New information:**

Here, we describe the SMTP dataset, published through the Global Biodiversity Information Facility (GBIF). Data on the sorted material are available in the "SMTP Collection Inventory" dataset. It currently includes more than 130,000 records of taxonomically-sorted samples. Data on the identified material are published using the Darwin Core standard for sample-based data. That information is divided up into group-specific datasets, as the sample set processed for each group is different and in most cases non-overlapping. The current data are divided into 79 taxonomic datasets, largely corresponding to taxonomic sorting fractions. The orders Diptera and Hymenoptera together comprise about 90% of the specimens in the material and these orders are mainly sorted to family or subfamily. The remaining insect taxa are mostly sorted to the order level. In total, the 79 datasets currently available comprise around 165,000 specimens, that is, about 1% of the total catch. However, the data are now accumulating rapidly and will be published continuously. The SMTP dataset is unique in that it contains a large proportion of data on previously poorly-known taxa in the Diptera and Hymenoptera.

## Introduction

Sweden has a long entomological tradition, starting even before Linnaeus's groundbreaking work on the Swedish fauna and flora. Nevertheless, large portions of the insect fauna remain poorly known to this day. The Swedish distribution of numerous species is documented only by scattered occurrence records and their ecology is poorly documented or unknown. It has also been clear for some time that further research on neglected insect groups would expand the known Swedish species stock significantly and lead to the discovery of a number of species new to science. These neglected groups include many taxa in the orders Diptera and Hymenoptera; it also includes the lice (Phthiraptera) and even some select groups in more well-known insect orders.

To address these knowledge gaps, an ambitious national insect inventory was started in 2003, the Swedish Malaise Trap Project (Karlsson et al. 2005, Karlsson et al. 2020). Malaise traps were chosen as the trapping method because of their efficiency in catching many of the poorly-known taxa of Diptera and Hymenoptera. A total of 73 Malaise traps were deployed at 55 localities spread out over the country and representing a diverse set of habitats. Most of the traps were run continuously from 2003 to 2006 or a significant portion of that time period. A detailed description of all accounts of the project, from collection through transfer to taxonomic experts and collection storage, was recently published (Karlsson et al. 2020).

The total SMTP catch is estimated to contain 20 million insects, distributed over 1,919 samples (Karlsson et al. 2020). The samples have been sorted into more than 300 taxonomic units, suitable for further processing by experts. Thus far, more than 100 taxonomists have been involved in identifying the sorted material, recording the presence of 4,000 species. One third of these had not been recorded from Sweden before and 700 have tentatively been identified as new to science (Ronquist et al. 2020).

The SMTP data are published through the Global Biodiversity Information Facility (GBIF). Data are published both on the sorted taxonomic fractions (the "SMTP Collection Inventory" dataset) and on the identified material of individual groups. The latter data are published using the Darwin Core standard for sample-based data to facilitate biodiversity analyses. The SMTP datasets are unique in that they contain a large proportion of data on previously poorly-known, but species-rich taxa in the Diptera and Hymenoptera. Due to this and the fact that the Swedish insect fauna was better known than most other insect fauna before the start of the inventory, the SMTP data offer biologists one of the best opportunities currently for detailed analysis of the size and composition of temperate insect fauna (Ronquist et al. 2020).

In this paper, we provide background data on the SMTP and describe the rationale behind the data publication strategy. We also provide an overview of the currently available datasets, comprising information on more than 130,000 taxonomically-sorted samples and on the species identity of around 165,000 specimens, that is, about 1% of the total SMTP catch. The species-level data are now accumulating rapidly; more than 600,000 specimens are currently on loan to experts for identification. The data will be published continuously through GBIF as they become available over the coming years.

## General description

### Purpose

The purpose of this paper is to provide an overview of the SMTP data published through GBIF and to describe the data publication strategy.

### Additional information

The SMTP data fall into two different categories: data on the material sorted to taxonomic fractions and data on the specimens identified to species. The data on the sorted material are available in the "SMTP Collection Inventory" dataset (Holston et al. 2020). It currently includes more than 130,000 records of taxonomically-sorted samples. As the taxonomic order sorting of the SMTP material is now complete, the changes to this dataset over the coming year or two will reflect only the finer levels of sorting as they occur. As samples are processed by taxonomists, however, they will disappear from the sorted material dataset so that it will continuously reflect the SMTP material that is currently available to taxonomic experts.

Data on the identified material are published using the Darwin Core standard for sample-based data. The goal is to make the data easy to use for biodiversity analyses, such as species richness estimation. Such analyses typically require that all species in a specific taxon set are recorded for the same set of samples. As the set of SMTP samples processed for each taxonomic unit is different, the information is divided into group-specific datasets. The taxon coverage of each dataset usually corresponds to one of the taxonomic fractions used in the sorting process. In some cases, several related taxonomic fractions are combined into a single dataset, but only if the same set of samples have been processed for all fractions. The circumscription of SMTP datasets is subject to change, based on discussions with the taxonomists involved in identifying the material, amongst other things.

The taxonomic coverage of each dataset is described in its metadata, in the taxonomic coverage section. The field generalTaxonomicCoverage is used to provide information about subtaxa that may be excluded. For instance, a genus may be excluded from the dataset of a family because it is very difficult to identify to species or because it is so numerous that identification to species would be too time-consuming. As appropriate for sample-based data, the absence of a species from the Occurrence table (the Extension table of the Darwin Core Archive) is significant. Provided that the species belongs to the covered taxon set, it means that the species was not encountered in the processed samples.

The samples processed for the taxa covered by the dataset are listed in the Event table (the Core table of the Darwin Core Archive). The sampling site location and sampling effort (time period in days) of each sample are specified, as well as the associated TrapID and EventID identifiers that are used consistently throughout the SMTP project. These identifiers facilitate analyses that look at patterns across SMTP datasets. For instance, one may be interested in the overlap in spatial or temporal coverage amongst SMTP datasets. The absence of one of the 1,919 samples from the Event table is significant: it means that the sample has not been processed for the taxa covered in the dataset. If a sample is listed in the Event table, but there are no occurrence records tied to it, it means that it has been verified that there are no specimens of the covered taxa in that sample. Note that the SMTP samples processed for the taxa in the dataset are usually an arbitrary subset of all the available SMTP samples. If the subset has been chosen according to a principled method, this will be noted in the metadata fields Methods:sampling and Methods:samplingDescription.

In general, the Occurrence table lists abundance data for the observed species. This may be recorded separately for each sex or as a total number of specimens regardless of sex. In a few cases, the Occurrence table instead lists incidence data or a mix of incidence and abundance data. If so, this is noted in the metadata of the dataset, in the Dataset:additionalInfo field. This field may also contain annotations about the determinations. For instance, in the Phoridae dataset, only the males are determined to species; females are usually determined only to genus.

As far as possible, the species-level taxonomy used for the SMTP data follows the national Swedish checklist Dyntaxa, also available as a checklist through GBIF (https://doi.org/10.15468/j43wfc). Deviations may occur for several reasons. Manuscript names are used in the SMTP datasets for new species; the ambition is to update these records and match them to new entries in Dynyaxa when the taxa are described. In some cases, specialists involved in identifying SMTP material provide corrections of Dyntaxa species names or concepts. These corrections are forwarded to Dyntaxa curators for review and possible action. The ambition is to synchronise the content in the SMTP datasets and Dyntaxa as soon as the issues have been resolved, but this is currently a manual process and time lags may occur.

The sample-based SMTP datasets are linked through their names; they are named "SMTP X", where "X" refers to the taxon set. For instance, the dataset covering Coleoptera is named "SMTP Coleoptera". The metadata of each dataset include standardised fields describing the SMTP project, further facilitating collective retrieval of all SMTP datasets.

## Project description

### Title

The Swedish Malaise Trap Project

### Personnel

The project was initially conceived by Fredrik Ronquist, then at Uppsala University and Thomas Pape, then at the Swedish Museum of Natural History. Project managers have been, from the beginning up to now: Johan Liljeblad, Kjell Arne Johanson, Kajsa Glemhorn and Dave Karlsson. The project was initially hosted by the Swedish Museum of Natural History, but the practical coordination of the project was moved to the field station Station Linné in 2006 and the project has been officially hosted by Station Linné since 2010. Questions about the project can be directed to Dave Karlsson, current SMTP project manager and corresponding author of this paper.

Traps were run largely by volunteers. Sorting has been carried out by staff, students and volunteers at Station Linné (for more details, see Karlsson et al. 2020). A large number of taxonomists (and students) have provided species identifications (see http://www.stationlinne.se/en/research/the-swedish-malaise-trap-project-smtp/taxonomic-units-in-the-smtp/ for updated lists). Crucial for handling the data on behalf of Station Linné have been Dave Karlsson, Pelle Magnusson, Mattias Forshage, Jessica Mo and Marina Karlsson and important for making them public on behalf of the Swedish Museum of Natural History have been Kevin Holston, Ida Li, Anders Telenius, Veronika Johansson, Manash Shah and Markus Skyttner.

### Study area description

**Figure 1.**
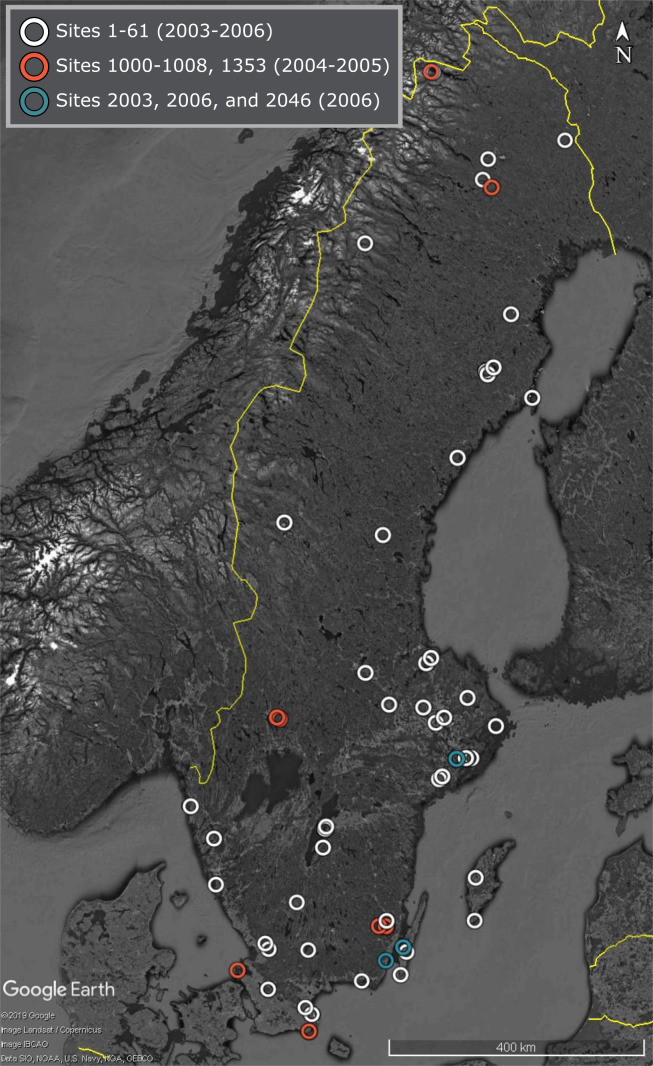
Map of SMTP trap sites colour coded by trapping period

A wide range of representative terrestrial habitats, distributed over Sweden (Fig. 1; Suppl. material 1)

### Design description

Sites were chosen to represent a broad range of habitats across the country of Sweden (Fig. 1, for more details, see Karlsson et al. 2020).

### Funding

The SMTP has been funded continuously since 2003 by the Swedish Taxonomy Initiative (Miller 2005, Ronquist and Gärdenfors 2003). Additional support for laboratory technicians has been obtained through the Swedish Public Employment Service and the Swedish Social Insurance Agency.

## Sampling methods

### Sampling description

**Figure 2.**
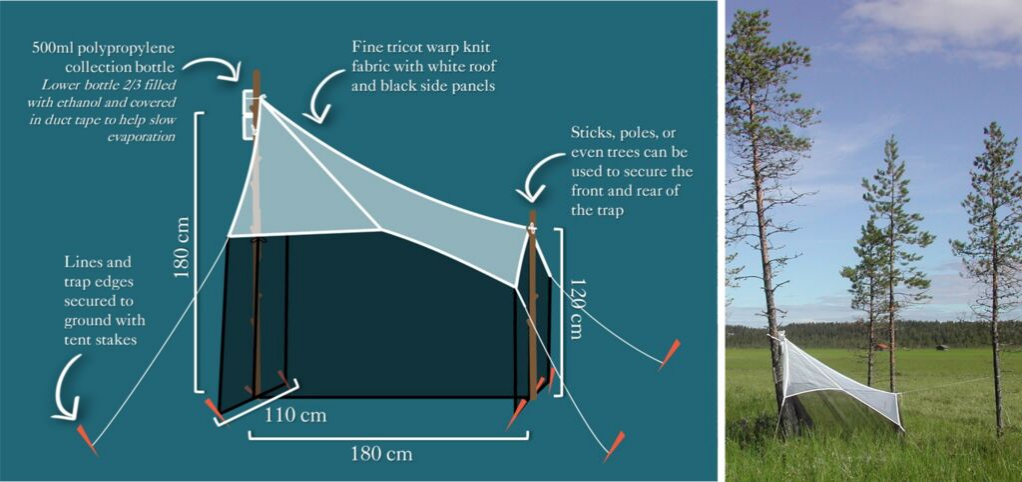
*Left*: Illustration of the Townes-style Malaise trap used by the SMTP. *Right*: A trap in operation at Norrbotten, Pajala Kommun (Site 49).

The project used a standard Townes-style Malaise trap obtained from Sante Traps, Lexington, KY, USA (Fig. 2).

The trap sites are described in more detail in Suppl. material 1. Trap locations were chosen to maximise habitat diversity (for more details, see Karlsson et al. 2020). To facilitate analyses, the sites have been classified into different habitat types (Suppl. material 1). A somewhat more detailed characterisation of habitat has been done by noting the dominant plants on each site. Suppl. material 2 provides a list of the 1,919 sampling events, each corresponding to a particular range of dates for a particular trap. Within the SMTP project, we maintain a unique set of identifiers for the traps and another set of identifiers for the collecting events. These identifiers are used consistently in all SMTP datasets to facilitate analyses that combine information from several datasets.

## Geographic coverage

### Description

The trap sites are spread throughout Sweden (Fig. 1; Suppl. material 1). Individual datasets of identified specimens span different subsets of the 1,919 samples and typically only include some of the trapping sites. Therefore, the geographic coverage varies considerably amongst datasets. As the geographic coverage will increase as data are added to each individual dataset, the actual geographic coverage of the dataset has to be computed from the Event table (the Core table in the Darwin Core Archive). The geographic coverage specified in the metadata of the dataset matches that of the entire project.

## Taxonomic coverage

### Description

The taxonomic coverage of the entire SMTP catch is quite broad. Malaise traps principally target flying insects (especially Hymenoptera and Diptera), but SMTP material also includes large numbers of other terrestrial arthropods (Araneae, Acari, Collembola) and scattered specimens of other invertebrates (Pulmonata, Lumbricidae etc). There are also single examples of unwanted bycatch of vertebrates (several lizards, a bird, a bat), but these specimens have not been preserved as part of the SMTP material.

The taxonomic coverage of each sample-based SMTP dataset is given as part of the metadata published with the dataset. The current data (Table 1, Suppl. material 3) comprise 79 different data datasets. The datasets are dominated by groups belonging to the orders Diptera and Hymenoptera, mostly at the family or subfamily level. Together, these orders comprise about 90% of the specimens in the SMTP material and they also dominate amongst the datasets that cover large numbers of samples and specimens (Table 1, Suppl. material 3). The remaining insect taxa in the SMTP material are mostly sorted to the order level; there are also substantial datasets for some of these. In total, the 79 data datasets currently available cover around 165,000 specimens, that is, about 1% of the total catch. The data are now accumulating rapidly and will be published continuously, so these numbers are likely to increase considerably over the coming years.

The datasets only cover a portion of the available taxonomic fractions from the SMTP material. About 330 taxonomic sorting fractions are currently used (Table 2; see the list at the Station Linné webpage for more details, including older sorting fractions). Around half of these fractions are at present actively being worked on. As new experts become involved in the project, we expect that the taxonomic coverage of the identified material will increase. The taxonomic fractions have changed slightly through the years, reflecting how experts want to work with the material at a given point in time and according to the sorting expertise available in the SMTP lab. These types of changes are likely to continue over the coming years. Similarly, we expect that the taxonomic circumscription of published datasets will change slowly over time.

### Taxa included

**Table:** 

Rank	Scientific Name	
class	Arachnida	
class	Entognatha	
class	Insecta	

## Temporal coverage

**Data range:** 2003-6-08 – 2006-11-23; 2007-4-11 – 2009-1-01.

### Notes

The primary collection phase started in the summer of 2003 and ended in late 2006 and involves 71 of the 73 traps at 53 locations. Single traps were run continuously throughout this period or through substantial parts of it (Karlsson et al. 2020). Two complementary traps were run at two additional sites from 11 April 2007 until 1 January 2009 (Suppl. material 2).

Trap managers were instructed to empty the traps every two weeks. However, during the most intense summer period, some traps had to be emptied more often. Conversely, during the winter, sample periods were often much longer (Suppl. material 2). Individual datasets of identified material span only a subset of the 1,919 samples and the temporal coverage therefore varies.

The temporal coverage of individual sample-based datasets varies considerably, depending on which samples have been processed. The temporal coverage will increase over time as data are added to the dataset. The actual temporal coverage has to be computed from the Event table (the Core table of the Darwin Core Archive); the metadata specify the temporal coverage of the entire project.

## Usage rights

### Use license

Creative Commons Public Domain Waiver (CC-Zero)

### IP rights notes

All SMTP datasets are released under the most permissive licence possible to facilitate use of the data.

## Data resources

### Data package title

SMTP DATA

### Resource link

SMTP Collection Inventory: https://doi.org/10.15468/5becml; SMTP Taxonomic Datasets (available as 79 datasets as published by Station Linné): https://www.gbif.org/publisher/bee30abf-e3a6-4e4e-b626-7ef57800b027

### Number of data sets

2

### Data set 1.

#### Data set name

SMTP Collection Inventory

#### Number of columns

20

#### 

**Data set 1.** 

Column label	Column description
Scientific name	Latin name of species
Country or Area	Location of specimen collection
Coordinates	Latitude and Longitude
Month & Year	Month and year of specimen collection
Basis of Record	All records based on preserved specimens
Dataset	The dataset containing the record
Individual count	The number of individual specimens recorded
Recorded by	All records by the Swedish Malaise Trap Project
Collection code	Unique identifier for collection
Institution code	Code of institution owning record/specimen
Identified by	Taxonomic expert who made identification
Publisher	All records published by the Swedish Malaise Trap Project
Rank	Rank of identification known
Kingdom	Level of identification
Phylum	Level of identification
Class	Level of identification
Order	Level of identification
Family	Level of identification
Genus	Level of identification
Species	Level of identification

### Data set 2.

#### Data set name

SMTP Taxonomic Dataset (broken into 79 subsets)

#### Number of columns

20

#### 

**Data set 2.** 

Column label	Column description
Scientific name	Latin name of species
Country or Area	Location of specimen collection
Coordinates	Latitude and Longitude
Month & Year	Month and year of specimen collection
Basis of Record	All records based on preserved specimens
Dataset	The dataset containing the record
Individual count	The number of individual specimens recorded
Recorded by	All records by the Swedish Malaise Trap Project
Collection code	Unique identifier for collection
Institution code	Code of institution owning record/specimen
Identified by	Taxonomic expert who made identification
Publisher	All records published by the Swedish Malaise Trap Project
Rank	Rank of identification known
Kingdom	Level of identification
Phylum	Level of identification
Class	Level of identification
Order	Level of identification
Family	Level of identification
Genus	Level of identification
Species	Level of identification

## Additional information

### Availability of material

The SMTP collection is maintained and curated as part of the insect collections of the Swedish Museum of Natural History (NRM) in Stockholm, Sweden. Most of the collection is kept in 95% ethanol at -20°C in modern storage facilities at Station Linné (see Karlsson et al. 2020 for details). Reference material identified by experts, as well as any type specimens, are deposited in the insect collections at the NRM.

### Data availability

The SMTP data will be continuously published through GBIF and we refer readers to GBIF (https://gbif.org) and the national Swedish hubs for natural history collections (https://naturarv.se) and biodiversity data (https://bioatlas.se), for the most recent versions of the datasets. General project information will be available from the Station Linné web site (https://stationlinne.se).

## Supplementary Material

8ED11BC1-F046-54AD-B650-75EB5FF622BB 10.3897/BDJ.8.e56286.suppl1 Supplementary material 1 Trapping site information Data type Site File: oo_452514.csv https://binary.pensoft.net/file/452514 Karlsson, D; Forshage, M; Holston, K; Ronquist, F

9157104B-ED8D-5ACC-971E-C0A980F3019C 10.3897/BDJ.8.e56286.suppl2 Supplementary material 2 Sampling events Data type Unique identifiers Brief description Each event corresponding to a particular range of dates for a particular trap File: oo_452524.xlsx https://binary.pensoft.net/file/452524 Karlsson, D; Forshage, M; Holston, K; Ronquist, F

AC589272-986A-5371-824C-BE1CE28CE7A7 10.3897/BDJ.8.e56286.suppl3 Supplementary material 3 The current 79 datasets of the SMTP Data type Occurrences Brief description Full version. File: oo_455180.xlsx https://binary.pensoft.net/file/455180 Karlsson, D; Forshage, M; Holston, K; Ronquist, F

## Figures and Tables

**Table 1.** The 79 current datasets of the SMTP. Full version is available as a supplementary file.

**Order**	**Dataset**	**Link to Dataset**	**Included Taxa**	Order
1	Odonata	https://doi.org/10.15468/5gtjxx	Odonata: all families	Odonata
2	Plecoptera	https://doi.org/10.15468/cvcqeb	Plecoptera: all families	Plecoptera
3	Dermaptera	https://doi.org/10.15468/w5xcbu	Dermaptera: all families	Dermaptera
4	Psocoptera	https://doi.org/10.15468/37b3pz	Psocoptera: all Psocodea except Phthiraptera	Psocoptera
5	Thysanoptera	https://doi.org/10.15468/8qm8rd	Thysanoptera: all families	Thysanoptera
6	Auchenorrhyncha excl Delphacidae	https://doi.org/10.15468/b48ufy	Hemiptera: Auchenorrhyncha: Cicadidae, Cicadellidae, Ulopidae, Membracidae, Aphrophoridae, Achilidae, Caliscelidae, Cixiidae, Issidae	Hemiptera
7	Psylloidea	https://doi.org/10.15468/cdw57a	Hemiptera: Sternorhyncha: Psylloidea	Hemiptera
8	Coleoptera	https://doi.org/10.15468/87hk5a	Coleoptera: all families	Coleoptera
9	Trichoptera	https://doi.org/10.15468/b3yrfu	Trichoptera: all families	Trichoptera
10	Microlepidoptera	https://doi.org/10.15468/wxyx7e	Lepidoptera: Micropteridoidea, Eriocranioidea, Hepialoidea, Nepticuloidea, Adeloidea, Tischerioidea, Tineoidea, Gracillarioidea, Douglasioidea, Yponomeutoidea, Gelechioidea, Tortricoidea, Schreckensteinioidea, Epermenioidea, Urodoidea, Choreutoidea, Alucitoidea, Pterophoroidea, Pyraloidea, Cossoidea	Lepidoptera
11	Macrolepidoptera	https://doi.org/10.15468/55t2ay	Lepidoptera: Zygaenoidea, Papilionoidea, Drepanoidea, Geometroidea, Lasiocampoidea, Bombycoidea, Sphingoidea, Noctuoidea	Lepidoptera
12	Strepsiptera	https://doi.org/10.15468/5ag9hn	Strepsiptera: all families	Strepsiptera
13	Mycetophilidae, Keroplatidae	https://doi.org/10.15468/dpa676	Mycetophilidae, Keroplatidae	Diptera
14	Small Sciaroidea families	https://doi.org/10.15468/stg5z9	Diadocidiidae, Mycetobiidae, Ditomyiidae, Sciarosoma	Diptera
15	Dixidae	https://doi.org/10.15468/u7dd6f	Dixidae	Diptera
16	Pachyneuridae	https://doi.org/10.15468/5epjpb	Pachyneuridae	Diptera
17	Cecidomyiidae: Porricondylinae (s lat)	https://doi.org/10.15468/hqpbmf	Cecidomyiidae: Porricondylinae, Winnertziinae	Diptera
18	Phoridae	https://doi.org/10.15468/ws6uh3	Phoridae	Diptera
19	Dolichopodidae	https://doi.org/10.15468/rs389z	Dolichopodidae	Diptera
20	Drosophilidae	https://doi.org/10.15468/9sjvbp	Drosophilidae	Diptera
21	Sepsidae	https://doi.org/10.15468/a6ugn5	Sepsidae	Diptera
22	Heleomyzidae, Odiniidae	https://doi.org/10.15468/gm4esc	Heleomyzidae	Diptera
23	Chloropidae	https://doi.org/10.15468/xqyhd8	Chloropidae	Diptera
24	Piophilidae	https://doi.org/10.15468/wchd9a	Piophilidae	Diptera
25	Sciomyzidae	https://doi.org/10.15468/bdzv7j	Sciomyzidae	Diptera
26	Lonchaeidae	https://doi.org/10.15468/8hsehm	Lonchaeidae	Diptera
27	Milichiidae excl Phyllomyza	https://doi.org/10.15468/ajpx9r	Milichiidae excl. Phyllomyza	Diptera
28	Muscidae	https://doi.org/10.15468/s5y2mb	Muscidae	Diptera
29	Anthomyzidae	https://doi.org/10.15468/zhb2jn	Anthomyzidae	Diptera
30	Tephritidae, Ulidiidae	https://doi.org/10.15468/y6dzym	Tephritidae, Ulidiidae	Diptera
31	Acartophthalmidae	https://doi.org/10.15468/nw85cf	Acartophthalmidae	Diptera
32	Lauxaniidae	https://doi.org/10.15468/q6653a	Lauxaniidae	Diptera
33	Trixoscelididae	https://doi.org/10.15468/7v7ybr	Trixoscelididae	Diptera
34	Pallopteridae	https://doi.org/10.15468/z244v6	Pallopteridae	Diptera
35	Asteiidae	https://doi.org/10.15468/z244v6	Asteiidae	Diptera
36	Syrphidae	https://doi.org/10.15468/4km26f	Syrphidae	Diptera
37	Dryomyzidae	https://doi.org/10.15468/9s6ktn	Dryomyzidae	Diptera
38	Conopidae	https://doi.org/10.15468/2denvh	Conopidae	Diptera
39	Aulacigastridae	https://doi.org/10.15468/n9qrv4	Aulacigastridae	Diptera
40	Clusiidae	https://doi.org/10.15468/2ng6hg	Clusiidae	Diptera
41	Asilidae	https://doi.org/10.15468/6d663p	Asilidae	Diptera
42	Therevidae	https://doi.org/10.15468/3n7h7p	Therevidae	Diptera
43	Symphyta	https://doi.org/10.15468/fag596	Xyelidae, Cephidae, Pamphiliidae, Siricidae, Xiphydriidae, Argidae, Blasticotomidae, Cimbicidae, Diprionidae, Tenthredinidae, Heptamelidae, Orussidae	Hymenoptera
44	Platygastridae (s str)	https://doi.org/10.15468/jh6duy	Platygastridae	Hymenoptera
45	Figitidae excl Charipinae	https://doi.org/10.15468/es2jyy	Figitidae: Anacharitinae, Aspicerinae, Eucoilinae, Figitinae	Hymenoptera
46	Ismaridae	https://doi.org/10.15468/zx6dxp	Ismaridae	Hymenoptera
47	Heloridae	https://doi.org/10.15468/wkxgbq	Heloridae	Hymenoptera
48	Evaniidae	https://doi.org/10.15468/t2nc3b	Evaniidae	Hymenoptera
49	Gasteruptiidae, Aulacidae	https://doi.org/10.15468/xknzj2	Gasteruptiidae, Aulacidae	Hymenoptera
50	Eupelmidae	https://doi.org/10.15468/y7ndas	Eupelmidae	Hymenoptera
51	Omphale	https://doi.org/10.15468/yvwnpu	Eulophidae: Entedoninae: Omphale	Hymenoptera
52	Mymar	https://doi.org/10.15468/sdgpvp	Mymaridae: Mymar	Hymenoptera
53	Ichneumoninae excl Phaeogenini	https://doi.org/10.15468/mer99q	Ichneumonidae: Ichneumoninae: Eurylabini, Goedartiinae, Heresiarchini, Ichneumonini, Listrodromini, Oedicephalini, Platylabini, Zimmeriini	Hymenoptera
54	Adelognathinae	https://doi.org/10.15468/f46vf7	Ichneumonidae: Adelognathinae	Hymenoptera
55	Diplazontinae	https://doi.org/10.15468/f9u8kc	Ichneumonidae: Diplazontinae	Hymenoptera
56	Pimplinae	https://doi.org/10.15468/8pu4y5	Ichneumonidae: Pimplinae	Hymenoptera
57	Rhyssinae	https://doi.org/10.15468/at6azc	Ichneumonidae: Rhyssinae	Hymenoptera
58	Poemeniinae	https://doi.org/10.15468/kq78ja	Ichneumonidae: Poemeniinae	Hymenoptera
59	Xoridinae	https://doi.org/10.15468/btczj6	Ichneumonidae: Xoridinae	Hymenoptera
60	Phrudus group	https://doi.org/10.15468/bt7w4p	Ichneumonidae: Tersilochinae: Phrudus group	Hymenoptera
61	Neorhacodes	https://doi.org/10.15468/saparq	Ichneumonidae: Neorhacodinae	Hymenoptera
62	Banchini	https://doi.org/10.15468/h8jaj2	Ichneumonidae: Banchinae: Banchini	Hymenoptera
63	Netelia	https://doi.org/10.15468/p3r2rb	Ichneumonidae: Tryphoninae: Netelia	Hymenoptera
64	Anomaloninae	https://doi.org/10.15468/4smbc7	Ichneumonidae: Anomaloninae	Hymenoptera
65	Brachycyrtinae	https://doi.org/10.15468/5zhs46	Ichneumonidae: Brachycyrtinae	Hymenoptera
66	Diacritinae	https://doi.org/10.15468/zycqbx	Ichneumonidae: Diacritinae	Hymenoptera
67	Cheloninae excl Adelius	https://doi.org/10.15468/68s836	Braconidae: Cheloninae excl. Adelius	Hymenoptera
68	Meteorini	https://doi.org/10.15468/c5fqds	Braconidae: Euphorinae: Meteorini	Hymenoptera
69	Rogadinae	https://doi.org/10.15468/jmn2xn	Braconidae: Rogadinae	Hymenoptera
70	Adelius	https://doi.org/10.15468/wg5p5e	Braconidae: Cheloninae: Adelius	Hymenoptera
71	Pompilidae	https://doi.org/10.15468/62qwjj	Pompilidae	Hymenoptera
72	Dryinidae, Embolemidae	https://doi.org/10.15468/3k4v3x	Dryinidae, Embolemidae	Hymenoptera
73	Small families of parasitic aculeates	https://doi.org/10.15468/d8xe2j	Mutillidae, Myrmosidae, Tiphiidae, Methochidae, Sapygidae, Pompilidae	Hymenoptera
74	Sphecidae (s lat)	https://doi.org/10.15468/bwhfe6	Crabronidae, Sphecidae, Ampulicidae	Hymenoptera
75	Vespinae	https://doi.org/10.15468/qck78f	Vespidae: Vespinae	Hymenoptera
76	Eumeninae	https://doi.org/10.15468/x656zx	Vespidae: Eumeninae	Hymenoptera
77	Formicidae	https://doi.org/10.15468/hd5npb	Formicidae	Hymenoptera
78	Chrysididae	https://doi.org/10.15468/g44yc3	Chrysididae	Hymenoptera
79	Bethylidae	https://doi.org/10.15468/633y44	Bethylidae	Hymenoptera

**Table 2.** Sorting fractions in the SMTP.

**First Tier Sorting Fraction**	**Second Tier Sorting Fraction**	**Third Tier Sorting Fraction**
ACARI		
AMPHIBIA		
AMPHIPODA		
ANNELIDA		
ARANEAE	Agelenidae	
ARANEAE	Amaurobiidae	
ARANEAE	Anyphaenidae	
ARANEAE	Araneidae	
ARANEAE	Argyronetidae	
ARANEAE	Atypidae	
ARANEAE	Clubionidae	
ARANEAE	Corinnidae (Syn: Phrurolithidae)	
ARANEAE	Dictynidae	
ARANEAE	Dysderidae	
ARANEAE	Eresidae	
ARANEAE	Gnaphosidae	
ARANEAE	Hahniidae	
ARANEAE	Linyphiidae	
ARANEAE	Liocranidae	
ARANEAE	Lycosidae	
ARANEAE	Mimetidae (Syn: Eutichuridae)	
ARANEAE	Miturgidae	
ARANEAE	Nesticidae	
ARANEAE	Oecobiidae	
ARANEAE	Oonopidae	
ARANEAE	Oxyopidae	
ARANEAE	Philodromidae	
ARANEAE	Pholcidae	
ARANEAE	Pisauridae	
ARANEAE	Salticidae	
ARANEAE	Segestriidae	
ARANEAE	Sparassidae (Heteropodidae)	
ARANEAE	Tetragnathidae	
ARANEAE	Theridiidae	
ARANEAE	Theridiosomatidae	
ARANEAE	Thomisidae	
ARANEAE	Titanoecidae	
ARANEAE	Uloboridae	
ARANEAE	Zoridae (Syn: Miturgidae)	
ARCHAEOGNATHA		
AUCHENORRHYNCHA		
AVES		
BLATTODEA		
BRACHYCERA	Acartophthalmidae	
BRACHYCERA	Acroceridae	
BRACHYCERA	Agromyzidae	
BRACHYCERA	Anthomyiidae	
BRACHYCERA	Anthomyzidae	
BRACHYCERA	Asilidae	
BRACHYCERA	Asteiidae	
BRACHYCERA	Athericidae	
BRACHYCERA	Aulacigasteridae	
BRACHYCERA	Bombyliidae	
BRACHYCERA	Braulidae	
BRACHYCERA	Calliphoridae	
BRACHYCERA	Camillidae	
BRACHYCERA	Campichoetidae	
BRACHYCERA	Carnidae	
BRACHYCERA	Chamaemyiidae	
BRACHYCERA	Chiropteromyzidae	
BRACHYCERA	Chloropidae	
BRACHYCERA	Chyromyidae	
BRACHYCERA	Clusiidae	
BRACHYCERA	Coelopidae	
BRACHYCERA	Coenomyiidae	
BRACHYCERA	Conopidae	
BRACHYCERA	Diastatidae	
BRACHYCERA	Dolichopodidae	
BRACHYCERA	Drosophilidae	
BRACHYCERA	Dryomyzidae	
BRACHYCERA	Empididae sensu lato (incl. Hybotidae, Atelestidae, Microphoridae, Brachystomatidae)	
BRACHYCERA	Empididae sensu stricto	
BRACHYCERA	Ephydridae	
BRACHYCERA	Fanniidae	
BRACHYCERA	Gasterophilidae	
BRACHYCERA	Helcomyzidae	
BRACHYCERA	Heleomyzidae (incl Borboropsidae)	
BRACHYCERA	Heterocheilidae (note: junior homonym)	
BRACHYCERA	Hippoboscidae	
BRACHYCERA	Hybotidae	
BRACHYCERA	Hypodermatidae	
BRACHYCERA	Lauxaniidae	
BRACHYCERA	Lonchaeidae	
BRACHYCERA	Lonchopteridae	
BRACHYCERA	Megamerinidae	
BRACHYCERA	Micropezidae	
BRACHYCERA	Milichiidae	
BRACHYCERA	Muscidae	
BRACHYCERA	Mythicomyiidae	
BRACHYCERA	Nycteribiidae	
BRACHYCERA	Odiniidae	
BRACHYCERA	Oestridae	
BRACHYCERA	Opetiidae	
BRACHYCERA	Opomyzidae	
BRACHYCERA	Pallopteridae	
BRACHYCERA	Periscelididae	
BRACHYCERA	Phaeomyiidae	
BRACHYCERA	Piophilidae	
BRACHYCERA	Pipunculidae	
BRACHYCERA	Platypezidae	
BRACHYCERA	Platystomatidae	
BRACHYCERA	Pseudopomyzidae	
BRACHYCERA	Psilidae	
BRACHYCERA	Rhagionidae	
BRACHYCERA	Rhinophoridae	
BRACHYCERA	Sarcophagidae	
BRACHYCERA	Scathophagidae	
BRACHYCERA	Scenopinidae	
BRACHYCERA	Sciomyzidae	
BRACHYCERA	Sepsidae	
BRACHYCERA	Sphaeroceridae	
BRACHYCERA	Stenomicridae	
BRACHYCERA	Stratiomyidae	
BRACHYCERA	Strongylophthalmyiidae	
BRACHYCERA	Syrphidae	
BRACHYCERA	Tabanidae	
BRACHYCERA	Tachinidae	
BRACHYCERA	Tanypezidae	
BRACHYCERA	Tephritidae	
BRACHYCERA	Tethinidae	
BRACHYCERA	Therevidae	
BRACHYCERA	Trixoscelididae	
BRACHYCERA	Ulidiidae	
BRACHYCERA	Xylomyidae	
BRACHYCERA	Xylophagidae	
BRACHYCERA : Phoridae		
COLEOPTERA		
COLLEMBOLA		
DERMAPTERA		
DIPLURA		
EPHEMEROPTERA		
GASTROPODA		
HETEROPTERA		
HYMENOPTERA	Apiformes (Anthophila)	
HYMENOPTERA	Aulacidae	
HYMENOPTERA	Bethylidae	
HYMENOPTERA	Braconidae	Adeliinae (Adelius)
HYMENOPTERA	Braconidae	Agathidinae
HYMENOPTERA	Braconidae	Alysiinae: Alysiini
HYMENOPTERA	Braconidae	Alysiinae: Dacnusini
HYMENOPTERA	Braconidae	Aphidiinae
HYMENOPTERA	Braconidae	Brachistinae: Blacini
HYMENOPTERA	Braconidae	Brachistinae:Brachistini
HYMENOPTERA	Braconidae	Braconinae
HYMENOPTERA	Braconidae	Cardiochilinae
HYMENOPTERA	Braconidae	Cenocoeliinae
HYMENOPTERA	Braconidae	Charmontinae
HYMENOPTERA	Braconidae	Cheloninae
HYMENOPTERA	Braconidae	Doryctinae
HYMENOPTERA	Braconidae	Euphorinae sensu stricto
HYMENOPTERA	Braconidae	Exothecinae
HYMENOPTERA	Braconidae	Gnamptodontinae
HYMENOPTERA	Braconidae	Helconinae sensu stricto
HYMENOPTERA	Braconidae	Histeromerinae
HYMENOPTERA	Braconidae	Homolobinae sensu stricto
HYMENOPTERA	Braconidae	Hormiinae
HYMENOPTERA	Braconidae	Ichneutinae
HYMENOPTERA	Braconidae	Lysiterminae
HYMENOPTERA	Braconidae	Macrocentrinae
HYMENOPTERA	Braconidae	Euphorinae: Meteorini
HYMENOPTERA	Braconidae	Microgastrinae
HYMENOPTERA	Braconidae	Microtypinae
HYMENOPTERA	Braconidae	Miracini
HYMENOPTERA	Braconidae	Euphorinae: Neoneurini
HYMENOPTERA	Braconidae	Opiinae
HYMENOPTERA	Braconidae	Orgilinae
HYMENOPTERA	Braconidae	Pambolinae
HYMENOPTERA	Braconidae	Proteropini
HYMENOPTERA	Braconidae	Rhysipolinae
HYMENOPTERA	Braconidae	Rhyssalinae
HYMENOPTERA	Braconidae	Rogadinae sensu stricto
HYMENOPTERA	Braconidae	Sigalphinae
HYMENOPTERA	Ceraphronidae	
HYMENOPTERA	Chalcidoidea sensu lato	Aphelinidae sensu lato (incl. Azotidae & Eriaporidae)
HYMENOPTERA	Chalcidoidea sensu lato	Chalcididae
HYMENOPTERA	Chalcidoidea sensu lato	Encyrtidae
HYMENOPTERA	Chalcidoidea sensu lato	Eulophidae
HYMENOPTERA	Chalcidoidea sensu lato	Eupelmidae
HYMENOPTERA	Chalcidoidea sensu lato	Eurytomidae
HYMENOPTERA	Chalcidoidea sensu lato	Mymaridae
HYMENOPTERA	Chalcidoidea sensu lato	Ormyridae
HYMENOPTERA	Chalcidoidea sensu lato	Perilampidae
HYMENOPTERA	Chalcidoidea sensu lato	Pteromalidae
HYMENOPTERA	Chalcidoidea sensu lato	Signiphoridae
HYMENOPTERA	Chalcidoidea sensu lato	Tetracampidae
HYMENOPTERA	Chalcidoidea sensu lato	Torymidae
HYMENOPTERA	Chalcidoidea sensu lato	Trichogrammatidae
HYMENOPTERA	ChalcidoideaMymaridae: Mymar	
HYMENOPTERA	Chrysididae	
HYMENOPTERA	Cynipoidea	Charipinae
HYMENOPTERA	Cynipoidea	Cynipidae
HYMENOPTERA	Cynipoidea	Figitidae (excl. Charipinae)
HYMENOPTERA	Cynipoidea	Ibaliidae
HYMENOPTERA	Diapriidae: Belytinae	
HYMENOPTERA	Diapriidae: Diapriinae	
HYMENOPTERA	Dryinidae	
HYMENOPTERA	Embolemidae	
HYMENOPTERA	Evaniidae	
HYMENOPTERA	Formicidae	
HYMENOPTERA	Gasteruptiidae	
HYMENOPTERA	Heloridae	
HYMENOPTERA	Ichneumonidae	Acaenitinae
HYMENOPTERA	Ichneumonidae	Adelognathinae
HYMENOPTERA	Ichneumonidae	Agriotypinae Haliday
HYMENOPTERA	Ichneumonidae	Alomyinae
HYMENOPTERA	Ichneumonidae	Anomaloninae
HYMENOPTERA	Ichneumonidae	Atrophini
HYMENOPTERA	Ichneumonidae	Banchinae
HYMENOPTERA	Ichneumonidae	Banchini
HYMENOPTERA	Ichneumonidae	Brachycyrtinae
HYMENOPTERA	Ichneumonidae	Campopleginae (Porizontinae)
HYMENOPTERA	Ichneumonidae	Collyriinae
HYMENOPTERA	Ichneumonidae	Cremastinae
HYMENOPTERA	Ichneumonidae	Cryptini Kirby
HYMENOPTERA	Ichneumonidae	Ctenopelmatini
HYMENOPTERA	Ichneumonidae	Cylloceriinae
HYMENOPTERA	Ichneumonidae	Diacritinae
HYMENOPTERA	Ichneumonidae	Diplazontinae
HYMENOPTERA	Ichneumonidae	Ephialtini excl. Polysphincta group (incl. Delomeristini and Perithoini)
HYMENOPTERA	Ichneumonidae	Eucerotinae
HYMENOPTERA	Ichneumonidae	Euryproctini
HYMENOPTERA	Ichneumonidae	Exenterini (incl. Eclytini)
HYMENOPTERA	Ichneumonidae	Glyptini
HYMENOPTERA	Ichneumonidae	Helictes group (≈Plectiscinae)
HYMENOPTERA	Ichneumonidae	Hemigastrini
HYMENOPTERA	Ichneumonidae	Hybrizontinae (Paxylommatinae)
HYMENOPTERA	Ichneumonidae	Ichneumonini sensu lato
HYMENOPTERA	Ichneumonidae	Idiogrammatini
HYMENOPTERA	Ichneumonidae	Lycorininae
HYMENOPTERA	Ichneumonidae	Mesochorinae
HYMENOPTERA	Ichneumonidae	Mesoleiini
HYMENOPTERA	Ichneumonidae	Metopiinae
HYMENOPTERA	Ichneumonidae	Microleptinae
HYMENOPTERA	Ichneumonidae	Neorhacodinae
HYMENOPTERA	Ichneumonidae	Oedemopsini
HYMENOPTERA	Ichneumonidae	Olethrodotini
HYMENOPTERA	Ichneumonidae	Ophioninae
HYMENOPTERA	Ichneumonidae	Orthocentrus group (Orthocentrini)
HYMENOPTERA	Ichneumonidae	Orthopelmatinae
HYMENOPTERA	Ichneumonidae	Oxytorinae
HYMENOPTERA	Ichneumonidae	Perilissini
HYMENOPTERA	Ichneumonidae	Phaeogenini
HYMENOPTERA	Ichneumonidae	Phrudini
HYMENOPTERA	Ichneumonidae	Phygadeuontini
HYMENOPTERA	Ichneumonidae	Phytodietini
HYMENOPTERA	Ichneumonidae	Pimplini
HYMENOPTERA	Ichneumonidae	Pionini
HYMENOPTERA	Ichneumonidae	Poemeniinae
HYMENOPTERA	Ichneumonidae	Polysphincta group (Polysphinctini)
HYMENOPTERA	Ichneumonidae	Rhyssinae
HYMENOPTERA	Ichneumonidae	Scolobatini
HYMENOPTERA	Ichneumonidae	Sphinctini
HYMENOPTERA	Ichneumonidae	Stilbopinae
HYMENOPTERA	Ichneumonidae	Tersilochinae sensu stricto
HYMENOPTERA	Ichneumonidae	Tryphonini
HYMENOPTERA	Ismaridae	
HYMENOPTERA	Megaspilidae	
HYMENOPTERA	Mutillidae	
HYMENOPTERA	Mymarommatoidea	
HYMENOPTERA	Myrmosidae	
HYMENOPTERA	Platygastridae	
HYMENOPTERA	Pompilidae	
HYMENOPTERA	Proctotrupidae	
HYMENOPTERA	Sapygidae	
HYMENOPTERA	Scelionidae	
HYMENOPTERA	Scoliidae	
HYMENOPTERA	Sparasionidae	
HYMENOPTERA	Spheciformes (Sphecidae sensu lato)	
HYMENOPTERA	Symphyta	Argidae
HYMENOPTERA	Symphyta	Blasticotomidae
HYMENOPTERA	Symphyta	Cephidae
HYMENOPTERA	Symphyta	Cimbicidae
HYMENOPTERA	Symphyta	Diprionidae
HYMENOPTERA	Symphyta	Heptamelidae
HYMENOPTERA	Symphyta	Tenthredinidae: Nematinae
HYMENOPTERA	Symphyta	Orussidae
HYMENOPTERA	Symphyta	Pamphiliidae
HYMENOPTERA	Symphyta	Siricidae
HYMENOPTERA	Symphyta	Tenthredinidae (excl. Nematinae)
HYMENOPTERA	Symphyta	Xiphydriidae
HYMENOPTERA	Symphyta	Xyelidae
HYMENOPTERA	Thynnidae	
HYMENOPTERA	Tiphiidae	
HYMENOPTERA	Vanhorniidae	
HYMENOPTERA	Vespidae	
ISOPODA Latreille, 1817		
LEPIDOPTERA		
LEPIDOSAURIA		
MAMMALIA		
"MECOPTERA"		
MEGALOPTERA		
MYRIAPODA		
"NEMATOCERA"	Anisopodidae	
"NEMATOCERA"	Bibionidae (incl Pleciidae)	
"NEMATOCERA"	Bolitophilidae	
"NEMATOCERA"	Canthyloscelidae (incl Synneuridae)	
"NEMATOCERA"	Cecidomyiidae	
"NEMATOCERA"	Ceratopogonidae	
"NEMATOCERA"	Chaoboridae	
"NEMATOCERA"	Chironomidae	
"NEMATOCERA"	Culicidae	
"NEMATOCERA"	Cylindrotomidae	
"NEMATOCERA"	Diadocidiidae	
"NEMATOCERA"	Ditomyiidae	
"NEMATOCERA"	Dixidae	
"NEMATOCERA"	Keroplatidae	
"NEMATOCERA"	Limoniidae	
"NEMATOCERA"	Mycetobiidae	
"NEMATOCERA"	Mycetophilidae	
"NEMATOCERA"	Pachyneuridae	
"NEMATOCERA"	Pediciidae	
"NEMATOCERA"	Psychodidae	
"NEMATOCERA"	Ptychopteridae	
"NEMATOCERA"	Scatopsidae	
"NEMATOCERA"	Sciaridae	
"NEMATOCERA"	Simuliidae	
"NEMATOCERA"	Thaumaleidae	
"NEMATOCERA"	Tipulidae	
"NEMATOCERA"	Trichoceridae	
ODONATA		
OPILIONES		
ORTHOPTERA sensu stricto (Saltatoria)		
PHTHIRAPTERA		
NEUROPTERA sensu stricto (Planipennia)		
PLECOPTERA		
PROTURA		
PSEUDOSCORPIONES		
"PSOCOPTERA"		
RAPHIDIOPTERA		
SIPHONAPTERA		
STERNORRHYNCHA		
STREPSIPTERA		
THYSANOPTERA		
TRICHOPTERA		
ZYGENTOMA (Thysanura sensu stricto)		

## References

[B5908707] Holston K, Karlsson D, Forshage M, Telenius A, Saha M, Johansson V (2020). Swedish Malaise Trap Project (SMTP) Collection Inventory.

[B3653553] Karlsson D, Pape T, Johanson KA, Liljeblad J, Ronquist F (2005). Svenska Malaisefälleprojektet, eller hur många arter steklar, flugor och myggor finns i Sverige?. Entomologisk Tidskrift.

[B5519432] Karlsson Dave, Hartop Emily, Forshage Mattias, Jaschhof Mathias, Ronquist Fredrik (2020). The Swedish Malaise Trap Project: A 15 year retrospective on a countrywide insect inventory. Biodiversity Data Journal.

[B5530960] Miller G. (2005). TAXONOMY: Linnaeus's legacy carries on. Science.

[B5531000] Ronquist Fredrik, Gärdenfors Ulf (2003). Taxonomy and biodiversity inventories: time to deliver. Trends in Ecology & Evolution.

[B5765350] Ronquist Fredrik, Forshage Mattias, Häggqvist Sibylle, Karlsson Dave, Hovmöller Rasmus, Bergsten Johannes, Holston Kevin, Britton Tom, Abenius Johan, Andersson Bengt, Buhl Peter Neerup, Coulianos Carl-Cedric, Fjellberg Arne, Gertsson Carl-Axel, Hellqvist Sven, Jaschhof Mathias, Kjærandsen Jostein, Klopfstein Seraina, Kobro Sverre, Liston Andrew, Meier Rudolf, Pollet Marc, Riedel Matthias, Roháček Jindřich, Schuppenhauer Meike, Stigenberg Julia, Struwe Ingemar, Taeger Andreas, Ulefors Sven-Olof, Varga Oleksandr, Withers Phil, Gärdenfors Ulf (2020). Completing Linnaeus's inventory of the Swedish insect fauna: Only 5,000 species left?. PloS one.

